# Jumonji domain-containing 6 (JMJD6) identified as a potential therapeutic target in ovarian cancer

**DOI:** 10.1038/s41392-019-0055-8

**Published:** 2019-07-26

**Authors:** Heng Zheng, Yan Tie, Zhen Fang, Xiaoai Wu, Tao Yi, Shuang Huang, Xiao Liang, Yanping Qian, Xi Wang, Ruyu Pi, Siyuan Chen, Yong Peng, Shengyong Yang, Xia Zhao, Xiawei Wei

**Affiliations:** 1 Department of Gynecology and Obstetrics, Key Laboratory of Obstetric and Gynecologic and Pediatric Diseases and Birth Defects of the Ministry of Education, West China Second Hospital, Sichuan University, 610041 Chengdu, P. R. China; 2 Lab of Aging Research and Cancer Drug Targets, State Key Laboratory of Biotherapy, West China Hospital, Sichuan University and Collaborative Innovation Center, No. 17, Block 3, Southern Renmin Road, 610041 Chengdu, Sichuan P. R. China

**Keywords:** Drug development, Gynaecological cancer

## Abstract

Jumonji domain-containing 6 (JMJD6) is a candidate gene associated with tumorigenesis, and JMJD6 overexpression predicts poor differentiation and unfavorable survival in some cancers. However, there are no studies reporting the expression of JMJD6 in ovarian cancer, and no JMJD6 inhibitors have been developed and applied to targeted cancer therapy research. In the present study, we found that the high expression of JMJD6 in ovarian cancer was correlated with poor prognosis in ovarian cancer. A potential inhibitor (SKLB325) was designed based on the crystal structure of the jmjC domain of JMJD6. This molecule significantly suppressed proliferation and induced apoptosis in a dose-dependent manner in SKOV3 cell lines as detected by CCK-8 cell proliferation assays and flow cytometry. A Matrigel endothelial tube formation assay showed that SKLB325 inhibited capillary tube organization and migration in HUVECs in vitro. We also observed that JMJD6 colocalized with p53 protein in the nucleus, with mRNA and protein expression of p53 as well as its downstream effectors significantly increasing both in vitro and in intraperitoneal tumor tissues treated with SKLB325. In addition, SKLB325 significantly reduced the intraperitoneal tumor weight and markedly prolonged the survival of tumor-bearing mice. Taken together, our findings suggest that JMJD6 may be a marker of poor prognosis in ovarian cancer and that SKLB325 may be a potential candidate drug for the treatment of ovarian cancer.

## Background

Ovarian cancer is the principal cause of death among gynecological malignant tumors.^1–3^ Mortality remains extraordinarily high, despite many studies on the mechanism of ovarian cancer^4^ and many improvements in treatments for patients with ovarian cancer, including surgery, radiotherapy, chemotherapy and new biological therapies.^5–7^ In addition, more than 70% of ovarian cancer patients are at advanced stages when initially diagnosed, and the 5-year survival rate is less than 30%.^8^ This terrible situation reflects a lack of novel, effective and feasible therapeutic approaches to treat ovarian cancer.

JMJD6, a member of the Jumonji C (JMJC) domain-containing family of proteins, which is a lysine hydroxylase,^9,10^ may regulate the transcriptional activity of p53 by hydroxylation of a lysine in the p53 C-terminal structural domain.^11–14^ Initially, JMJD6 was studied as the phospholipid methionine receptor on the surface of phagocytic cells.^15–17^ Subsequently, many studies showed that JMJD6 has multiple nuclear positioning signals and a JMJC domain, indicating that it might have a novel function in the nucleus.^18–22^ Recent studies indicate that JMJD6 is present in various organisms^20^ and is mainly located in the nucleus.^16,21–25^ Its protein sequence contains JMJC folds,^26,27^ which are double-stranded β-helix or typical cupin folds shared by all 2-oxopentane acid-dependent dioxygenases (2-OG),^28,29^ and catalytic studies also identified JMJD6 as a functional 2-OG oxygenase.^30,31^ Elevated levels of some tricarboxylic acid cycle intermediates can inhibit 2-OG oxygenases in some tumors.^32,33^ The Fe^2+^ binding site of the catalytic center of JMJD6 is located at the opening of the double-stranded beta-helix fold, forming a barrel structure, which is important for the enzymatic activity of JMJD6.^26,27,34,35^ The demethylation of dimethylated arginine residues was shown to be catalyzed by JMJD6 on histone H3 arginine 2 (H3R2me2) and histone H4 arginine 3 (H4R3me2).^27,34^ However, the current consensus is that JMJD6 is a lysyl 5-hydroxylase, not a demethylase.^10,26,36^ Studies also demonstrated that JMJD6 interacts with bromodomain-containing protein 4 (Brd4), suggesting that JMJD6 and Brd4 are joined together on anti-pause enhancers (A-PEs).^34^ The ablation of the JMJD6 gene in *Caenorhabditis elegans* delayed the engulfment of apoptotic cells,^15,37^ and depletion of JMJD6 promoted apoptosis, induced cell death by sensitizing cells to DNA damaging reagent, and repressed cell tumorigenesis and proliferation, which is mediated by p53.^9^ Furthermore, some studies have suggested that the high expression of JMJD6 is related to tumor growth, tumor metastasis and high tumor pathological classification.^9,38–41^

Recent studies indicate that JMJD6 may play a role in other reactions, such as inflammation, infections and immune responses. These reports showed a decrease in the expression of JMJD6 in T lymphocytes of patients who had chronic hepatitis B virus infection,^42^ and the proliferation of CD4+ T cells could be inhibited by reducing the expression of JMJD6 in normal peripheral blood mononuclear cells.^42,43^ For the expression of JMJD6 in immune cells and its role in RNA metabolism, replication of viral RNA may be necessary.^44,45^

Recent studies also indicate that expression of JMJD6 in some human cancers, such as lung adenocarcinoma,^39^ breast ductal carcinomas,^40^ and colon adenocarcinomas,^9^ is evidently upregulated. These data indicate that JMJD6 may be an attractive therapeutic target for cancer intervention. However, the expression of JMJD6 in ovarian cancer has not been reported. Additionally, no JMJD6 inhibitors have been developed and applied to targeted cancer research. In our research, the expression of JMJD6 was detected by tissue microarray immunohistochemical staining, and we found high expression of JMJD6 in ovarian cancer, which was correlated with poor prognosis. Furthermore, we designed inhibitors based on the crystal structure of the jmjC domain of JMJD6 and selected the most active inhibitor, SKLB325, and conducted a study of the antitumor effects of SKLB325 on ovarian cancer in vivo and in vitro.

## Methods

### Human ovarian tissue samples

**Scheme 1:**
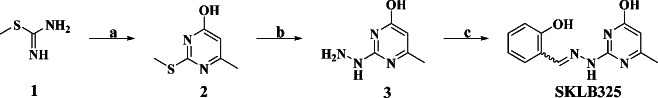
The synthetic route for SKLB325. Reagents and conditions: **a** ethyl acetoacetate, Na_2_CO_3_, H_2_O, rt, overnight; **b** hydrazine hydrate, K_2_CO_3_, isopropanol, reflux, overnight; **c** 2-hydroxybenzaldehyde, methanol, reflux, overnight

JMJD6 expression in 151 patients with ovarian masses who underwent surgical excision between February 2009 and February 2013 was analyzed, including two borderline ovarian tumor tissues, three para-carcinoma tissues, 14 ovarian cancer metastasis tissues, and 132 primary ovarian cancer tissues (Scheme 1). None of these patients received preoperative radiotherapy or chemotherapy. All patients were followed up for at least 5 years after the operation. Pathological diagnosis and staging of tumors was in accordance with WHO and FIGO criteria.^2^ Sections were incubated with anti-JMJD6 monoclonal mouse antibody (Santa Cruz Biotechnology, sc-28348, 1:50). Then, slices were incubated with biotinylated secondary antibodies and a third antibody. Diaminobenzidine was used for detecting colorimetric activity. Negative controls were included by omitting the primary antibody. Five random fields of view at ×200 were examined for each tissue. Referring to the previous literature for the classification of JMJD6 expression,^41^ our staining results were scored as follows: low staining (1+), <10% of tumor cells positively stained; intermediate staining (2+), 10–80% of tumor cells with positive staining; strong staining (3+), positive staining in >80% of tumor cells. The samples with 1+ and 2+ staining were defined as the “low expression group,” and those with 3+ staining were classified as the “high expression group.”

### Chemistry

All materials and reagents used in our study were purchased from commercial vendors without further purification. A Bruker AV-400 spectrometer was used to record ^13^C NMR and ^1^H NMR spectra at 400 and 100 MHz, respectively. Thin layer chromatography (TLC) was used for monitoring reactions, and it was performed on Merck silica gel 60 F-254 thin layer plates. High-performance liquid chromatography (HPLC) was used for determining final compounds whose purities were all >95%. All the determinations were performed on a Waters 2695 HPLC system equipped with a Kromasil C18 column (4.6 mm × 250 mm, 5 μm).

Ethyl acetoacetate (10 g, 77 mmol) and 6-methyl-2-(methylthio) pyrimidin-4-ol (2): sodium carbonate (16.29 g, 154 mmol) were added to a solution of 2-methyl-2-thiopseudourea sulfate (15.91 g, 85 mmol) in water (200 mL) at room temperature and then churned overnight. After neutralizing to pH = 8, filtrating, and drying under vacuum overnight, the solid was collected. The product was used without further purification.

For 2-hydrazinyl-6-methylpyrimidin-4-ol (3): To a suspension of 6-methyl-2-(methylthio) pyrimidin-4-ol (2.1 g, 13 mmol) and potassium carbonate (140 mg, 1 mmol) in 2-propanol (40 mL), hydrazine hydrate (6.3 mL, 130 mmol) was added dropwise at room temperature. After cooling to room temperature, the precipitate was filtered, washed with methanol, and dried under vacuum overnight. The product was used without further purification.

For 2-(2-(2-hydroxybenzylidene) hydrazinyl)-6-methylpyrimidin-4-ol (SKLB325): A suspension of 2-hydrazinyl-6-methylpyrimidin-4-ol (1.8 g, 13 mmol) and 2-hydroxybenzaldehyde (3.14 g, 26 mmol) in methanol (30 mL) was heated at reflux overnight. ^1^H NMR (400 MHz, DMSO-*d*_6_) δ 11.26 (br s, 2 H), 9.98 (br s, 1 H), 8.36 (s, 1 H), 8.04 (d, *J* = 7.8 Hz, 1 H), 7.29–7.14 (m, 1 H), 6.89–6.82 (m, 2 H), 5.52 (s, 1 H), 2.08 (s, 3 H). ^13^C NMR (101 MHz, DMSO-*d*_6_) δ 163.15, 156.54, 152.82, 143.04, 131.26, 128.42, 120.79, 119.66, 116.46, 101.53, 23.08. ESI-ms (m/z):245.1[M+H]^+^.

### SPR analysis

Biacore T200 instruments were used for the surface plasmon resonance (SPR) procedure at 25 °C. BIA evaluation software (GE Healthcare) was used to determine the *K*_D_ value of the compounds.

### Drug concentration

The inhibitor (SKLB325) powder was completely dissolved in DMSO at a concentration of 40000 μM.

### Cell lines and cell culture

The cell lines SKOV3, ES2, CP70, and A2780s used in this study were purchased from the American Type Culture Collection (ATCC, Manassas, VA) and were cultured in Dulbecco’s modified Eagle’s medium (DMEM) supplemented with 10% heat-inactivated fetal bovine serum (FBS), 100 U/ml penicillin, 0.1 mg/ml streptomycin and 2 mmol L-glutamine. DMEM and FBS were purchased from Gibco. Human umbilical vein endothelial cells (HUVECs) were supported by the Department of Obstetrics & Gynecology, West China Second University Hospital, Sichuan University, Chengdu, China and were isolated from human umbilical cord. Cells used in our study were incubated at 37 °C in a humidified circumstance containing 5% CO_2_. Trypsin was used for passaging cells at a ratio of 1:3 every 3 days.

### CCK-8 cell proliferation assay

SKOV3 cells were seeded in 96-well plates at 3000–5000 cells/well in 100 μl of medium and cultured for 24, 48, or 72 h. After incubation periods, 100 μl of DMEM containing 10% Cell Counting Kit-8 (CCK-8) (Beyotime, China) was added to each well. To form water-insoluble formazan, cells were incubated for another 3 h at 37 °C. A microplate reader was used to measure the absorbance at 450 nm according to the instructions. Each concentration was replicated in at least four wells. Vehicle, control, and blank groups were created simultaneously: the vehicle group contained cells with DMSO concentrations equal to that of corresponding drug wells and 10% CCK-8; the control group contained cells and 10% CCK-8; the blank group contained only 10% CCK-8. Data for inhibition were calculated by the following formula: Inhibition = (SKLB325- vehicle)/(control-blank), viability = 1-inhibition.

### Confocal microscopy

The slides of SKOV3 cells were imaged after immunofluorescence staining, which was performed with JMJD6 (Abcam, 1:50) and p53 (Santa Cruz, 1:50) primary antibodies. Alexa Fluor 488-conjugated goat-anti-rabbit antibody (Invitrogen, 1:100) and Alexa Fluor 594-conjugated goat-anti-rat antibody (Invitrogen, 1:100) were used as the secondary antibodies. Then, the cells were stained with 5 μg/ml DAPI for 10 min, followed by washing and imaging using a Leica TCS SP5 confocal microscope. SKOV3 cells stained with secondary antibodies but without primary antibodies were used as the negative controls.

### Matrigel endothelial tube formation assay

Matrigel (BD, USA) was melted at 4 °C overnight, and 100 μl of Matrigel/well was vertically plated into 24-well plates and cultured at 37 °C for 45 min. Then, 1 × 10^5^ HUVECs and 4 μM of SKLB325 were added into each well simultaneously with cultured Matrigel. DMSO in media was used as a control. After culturing at 37 °C for 4 h, a phase contrast microscope (OLYMPUS inverted digital camera) was used to observe the tube formation, and the number of tubes was counted manually in five random microscopic fields at ×100 magnification.

### Transwell migration assay

A total of 4 × 10^4^ HUVECs, SKOV3, A2780s and ES2 cells were resuspended in 200 μL DMEM medium with 4 μM of the inhibitor and placed in the upper chamber. DMSO in media was used as a control. Then, the chamber was placed in a 24-well plate containing 600 μL DMEM and 10% FBS. After incubation at 37 °C overnight, migrating cells were counted manually in five random microscopic fields at ×200 magnification.

### Quantitative real-time reverse transcription PCR

An RNA Simple Total RNA Kit (Tiangen Biotech, China) was used for preparing total cellular RNA, and a PrimeScript RT reagent Kit with gDNA Eraser (TaKaRa) was used for reverse transcribing isolated RNA into cDNA. Sso Advanced SYBR Green Supermix (Bio-Rad) was used for quantifying all gene transcripts by qPCR with a two-step PCR procedure. A CFX96 Real-Time System (Bio-Rad) was used to measure mRNA levels. Expression of GAPDH served as the internal control. Sense and antisense primers were as follows: 5′-TTTAACTCTGGTAAAGTGGA-3′ and 5′-GAATCATATTGGAACATGTA-3′ for GAPDH; 5′-GACGACCTCAACGCACAGTA-3′ and 5′-CACCTAATTGGGCTCCATCT-3′ for PUMA; 5′-GGACCTGGAGACTCTCA-3′ and 5′-CCTCTTGGAGAAGATCA-3′ for p21; 5′-GTTCCGAGAGCTGAATGAGG-3′ and 5′-TCTGAGTCAGGCCCTTCTGT-3′ for p53.

### Apoptosis assay

Aliquots of 5 × 10^4^ cells were plated in six-well plates in triplicate. After 4 h, we changed the medium to 2 ml DMEM containing 10% fetal bovine serum and a series of drug concentrations as well as corresponding doses of DMSO. Cells were incubated for 72 h to 80% confluence, at which time both the attached cells and floating cells were trypsinized, collected in flow cytometry tubes and washed twice with cold PBS. Cells were resuspended in 100 μl of 1x binding buffer and stained with FITC-Annexin V and PI (BD Pharmingen, CA) for 10 min in the dark and then analyzed by flow cytometry (Novoexpress, China).

### Western blot analysis

Samples from each group were lysed in modified RIPA lysis buffer (Beyotime Biotech, China), which contained protease inhibitor cocktail (Sigma), and then placed on ice for 30 min. Then, samples were centrifuged at 13000 rpm and 4 °C for 30 min. Protein concentrations were determined using a BCA protein assay kit (Pierce, Thermo Fisher Scientific, Inc., Waltham, MA, USA). Equal quantities of protein were loaded onto 12.5% SDS-PAGE for electrophoresis, followed by transfer to PVDF membranes (Millipore). After blocking with 5% nonfat milk for 2 h, membranes were immunoblotted with rabbit anti-human polyclonal antibodies against p53 (1:5000, Santa Cruz), p21 (1:1000, CST) and PUMA (1:1000, Abcam) overnight at 4 °C. Then, appropriate secondary antibodies (goat-anti-rabbit antibody, 1:10000; ZSGB-BIO, Beijing, China) were used for incubating the blots for 1 h at 37 °C. GAPDH was used as a control (1:5000; Zen-BioScience, Chengdu, China). A western blotting luminol reagent (Millipore, MA, USA) was used to visualize the immunoreactive bands.

### Establishment of a peritoneal carcinomatosis model in BALB/c nude mice

Female athymic BALB/c nude mice used in this study were purchased from HFK Bioscience (Beijing, China) at 5–6-weeks-old. The mice were housed in microisolator cages and fed sterilized water and food.

Five nude mice were used for subcutaneous (s.c.) injection with 5 × 10^6^ SKOV3 cell suspensions in their right backs. When the tumor diameter reached approximately 1 cm, tumors were minced into small particles within a diameter of 1 mm after being isolated free of normal and necrotic tissues. DMEM medium was used to resuspend these tumor tissues in a total volume of 10 ml. Then, 0.5 ml of the resuspension was inoculated i.p. in the right lower quadrant of fifteen nude mice with a 14-gauge needle. As in previous studies performed in our laboratory, as early as 7 days after i.p. inoculation, tumor nodules could be observed, and extensive dissemination of intraperitoneal carcinomatosis was also detected. For this, 7 days after injection of tumor particles, therapeutic studies were started.

### Therapy studies

First, the drug was prepared (mixture of 20 μl of SKLB325 ultimate solution and 80 μl of normal saline (NS)). As a control for our drug, equal concentrations of DMSO were used in the vehicle group.

The mice mentioned above were randomly assigned into three groups (*n* = 5): (1) untreated mice, (2) mice treated with 100 μl of NS and DMSO, or (3) mice treated with 100 µl of NS and SKLB325 (10 mg/kg). Mice received i.p. injections every three days for eight doses total and were monitored for abdominal distension, tumor burden and other pathologies before i.p. administration. Three days after the last administration, all fifteen mice were sacrificed, and tumors were subsequently isolated, counted and weighed. We also used five nude mice without nodules as the normal control group from the same batch as the mice in the three experimental groups.

To detect the effect of our compound against ovarian cancer, we observed the survival time as previously described. When mice were at dying status, they were sacrificed, and we recorded the day when they were sacrificed as their survival time.

### Immunohistochemistry

After fixing the intraperitoneal tumors in 4% paraformaldehyde all night, we embedded them in paraffin and cut the wax blocks into 3–4 mm slices. For antigen retrieval, slices were dewaxed first, after which the slices were immersed in 10 mmol/L sodium citrate buffer (pH 6.0), followed by heating in an autoclave that was in saturated conditions for 3 min. Then, 3% H_2_O_2_ was used for quenching the endogenous peroxidase activity, the procedure was performed in a dark room at room temperature for 10 min, and normal goat serum was used for blocking the nonspecific binding at 37 °C for 20 min. Next, the slices were incubated with primary antibodies against P53, PUMA, and Ki-67 at 4 °C overnight. Then, slices were incubated with biotinylated secondary antibodies at 37 °C for 40 min; subsequently, they were incubated with a third antibody at 37 °C for 40 min. Diaminobenzidine was used for the colorimetric detection. Sections of the intraperitoneal tumours were used to detect vessel density with anti-VEGF and anti-CD31 antibodies, of which CD31 antibody was used for frozen sections. Negative controls were included by omitting primary antibody.

### In situ TUNEL

Apoptosis in human ovarian cancer xenograft tumours was examined using a TUNEL kit (Merck Millipore) following the manufacturer’s instructions. Five tumours per group were analyzed. Fluorescence microscopy was used to quantify the number of TUNEL-positive cells, and the apoptotic index in at least five random fields per group at ×400 magnification was calculated.

### Toxicity evaluation

Diarrhea, weight loss, ruffling of fur, anorexia, behavioral changes, and even deaths as relevant indexes of another 15 mice were observed continuously to detect potential toxicity to mice in response to treatment in our study. In addition, sections of heart, lung, liver, spleen, and kidney tissues were stained with hematoxylin and eosin (H&E) and histologically evaluated by professional pathologists. In addition, routine analysis and biochemical analysis of blood was performed for every mouse.

### Statistical analysis

The relationship between JMJD6 expression and clinicopathological characteristics was analyzed by Pearson’s *X*^2^ test. Overall survival (OS), defined as time from operation to death or date of last follow-up, disease-free survival (DFS), defined as time from operation to relapse, and death or date of last follow-up were studied. Survival curves were analyzed by the Kaplan–Meier method, and the log-rank test was used for comparing groups. Statistical analysis was performed using IBM SPSS Statistics 23. A *P* value < 0.05 was considered to be statistically significant.

## Results

### The expression of JMJD6 is correlated with some clinicopathologic characteristics

**Fig. 1:**
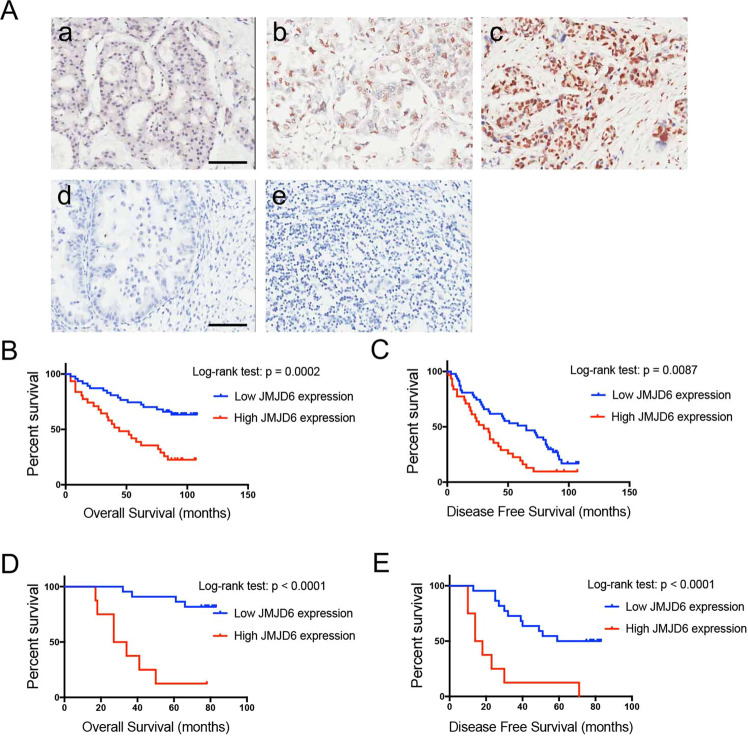
Kaplan–Meier estimates of OS and DFS according to JMJD6 expression groups. **a** JMJD6 protein expression in ovarian cancer tissues detected by immunohistochemistry analysis. a Low staining, b Intermediate staining, c Strong staining of JMJD6 protein, d Negative control (normal adjacent tissues of ovarian cancer), e Primary antibody-free negative control, magnification, ×200. **b** Overall survival of serous ovarian cancer, log-rank test: *p* = 0.0002. **c** Disease-free survival of serous ovarian cancer, log-rank test: *p* = 0.0087. **d** Overall survival of mucinous ovarian cancer, log-rank test: *p* < 0.0001. **e** Disease-free survival of mucinous ovarian cancer, log-rank test: *p* < 0.0001

The expression of JMJD6 protein in 146 ovarian cancer tissue samples was detected by immunohistochemical staining to explore the relationship between the expression of JMJD6 and the clinicopathological characteristics of ovarian cancer patients. As shown in Fig. 1a and Table 1, JMJD6 was highly and lowly expressed in 90 (61.64%) and 56 (38.36%) of the 146 ovarian cancer patients, respectively, according to the intensity of immunoreaction. As shown in Table 1, high expression of JMJD6 was significantly associated with age (*p* = 0.026), clinical stage (*p* < 0.001), pT status (*p* = 0.015), pN status (*p* = 0.003) and relapse (*p* = 0.002). However, no significant relationship was found between JMJD6 protein expression and characteristics such as tumor diameter (*p* = 0.140), pathological grade (*p* = 0.136) and pM status (*p* = 0.765).

**Table 1:** Association between JMJD6 expression and clinicopathological variables of ovarian cancer patients

Variables	No.	JMJD6 protein expression		*P* value
		Low	High	
Age	146	90	56	0.026
<60	111	74	37	
≥60	35	16	19	
Tumor diameter	146	90	56	0.140
<10 cm	58	40	18	
≥10 cm	88	50	38	
Pathological grade	118	69	51	0.136
1	13	10	3	
2–3	105	58	47	
Clinical stage	146	90	56	<0.001
I–II	45	38	7	
III–IV	101	52	49	
pT status	146	90	56	0.015
pT 1	9	9	0	
pT 2–3	137	81	56	
pN status	146	90	56	0.003
pN 0	106	73	33	
pN 1	40	17	23	
pM status	146	90	56	0.765
pM 0	114	71	43	
pM 1	32	19	13	
Relapse	146	90	56	0.002
Yes	116	64	52	
No	30	26	4	

*No* number

### JMJD6 is a marker of poor prognosis in ovarian cancer

We next investigated the prognosis of JMJD6 expression in ovarian cancer. A cohort of 146 ovarian cancer samples was detected by immunohistochemical staining. We found that the nuclear staining of JMJD6 varied from low levels (Fig. 1a, a) to intermediate (b) and strong levels (c) in ovarian cancer tissue. In addition, normal adjacent tissues of ovarian cancers were used for the negative controls (Fig. 1a, d), and primary antibody-free staining of ovarian cancer tissues was used for the primary antibody-free negative controls (Fig. 1a, e). JMJD6 was highly expressed in 31 (39.74%) of the 78 serous ovarian cancer patients and in eight (26.67%) of the 30 mucinous ovarian cancer patients. Kaplan–Meier survival analysis indicated that serous and mucinous ovarian cancer patients with high JMJD6 expression had a significantly shorter median overall survival as well as disease-free survival (*p* = 0.0002, *p* = 0.0087, *p* < 0.0001, and *p* < 0.0001, respectively, Fig. 1b–e). Kaplan–Meier survival analysis also showed that patients with high JMJD6 expression had a significantly shorter median overall survival and disease-free survival (*p* < 0.0001 and *p* = 0.0001).

As shown in Tables 2 and 3, in terms of patient outcome, univariate Cox regression analysis indicated that JMJD6 expression (*p* < 0.001 and *p* < 0.001), age (*p* = 0.003 and *p* = 0.001), pT status (*p* < 0.001 and *p* < 0.001), pN status (p < 0.001 and *p* < 0.001), and pM status (*p* < 0.001 and *p* < 0.001) were significantly associated with OS as well as DFS. Furthermore, multivariate Cox regression analysis was used to determine whether the expression of JMJD6 could be an independent predictor for OS or DFS of ovarian cancer. The results revealed that expression of JMJD6 (*p* = 0.012), pT (*p* = 0.002) status and pM (*p* = 0.003) status remained independent prognostic factors for OS, although no significant association was found for JMJD6 as an independent prognostic factor for DFS (*p* = 0.232) (Tables 2 and 3). Altogether, these data showed that expression of JMJD6 may play a role in predicting OS in ovarian cancer.

**Table 2:** Univariate and multivariate analysis showing the OS rate for ovarian cancer patients

Variables	Univariate analysis			Multivariate analysis		
	RR	95% CI	*P* value	RR	95% CI	*P* value
JMJD6: % of stained cells	2.955	1.879–4.646	<0.001	2.005	1.169–3.438	0.012
Age	1.031	1.011–1.051	0.003	1.009	0.987–1.031	0.411
pT status	0.098	0.036–0.269	<0.001	0.131	0.036–0.482	0.002
pN status	5.807	3.656–9.226	<0.001	1.113	0.559–2.216	0.76
pM status	6.226	3.823–10.139	<0.001	2.935	1.427–6.04	0.003
Tumor diameter	0.778	0.354–1.711	0.533	0.692	0.307–1.558	0.373
Pathological grade	0.376	0.092–1.535	0.173	3.17	0.623–16.141	0.165

*RR* relative risk, *95% CI* 95% confidence interval

**Table 3:** Univariate and multivariate analysis showing the DFS rate for ovarian cancer patients

Variables	Univariate analysis			Multivariate analysis		
	RR	95% CI	*P* value	RR	95% CI	*P* value
JMJD6: % of stained cells	2.783	1.769–4.377	<0.001	1.385	0.812–2.364	0.232
Age	1.032	1.012–1.052	0.001	1.008	0.988–1.029	0.433
pT status	0.09	0.033–0.246	<0.001	0.107	0.027–0.425	0.001
pN status	5.698	3.587–9.053	<0.001	1.178	0.605–2.295	0.63
pM status	6.276	3.849–10.233	<0.001	2.449	1.242–4.831	0.01
Tumor diameter	0.935	0.426–2.054	0.868	0.777	0.344–1.757	0.545
Pathological grade	0.352	0.086–1.44	0.146	2.876	0.519–15.923	0.226

*RR* relative risk, *95% CI* 95% confidence interval

### Virtual screening to retrieve new potential inhibitors for JMJD6

**Fig. 2:**
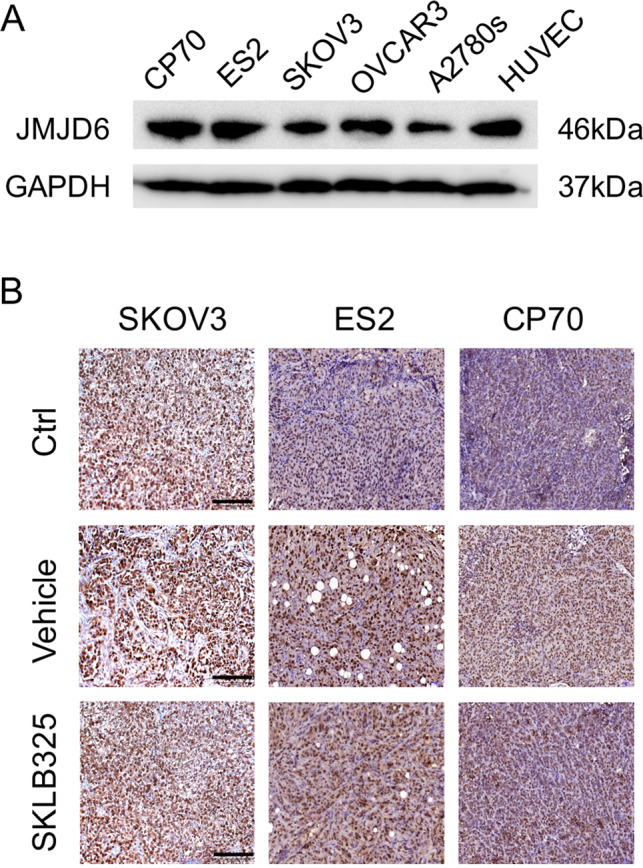
Expression of JMJD6 in vitro and in vivo. **a** JMJD6 is highly expressed in ovarian cancer cells and HUVECs, which was examined by western blot. **b** JMJD6 is highly expressed in tumor tissues isolated from an intraperitoneal dissemination xenograft mouse model

**Fig. 3:**
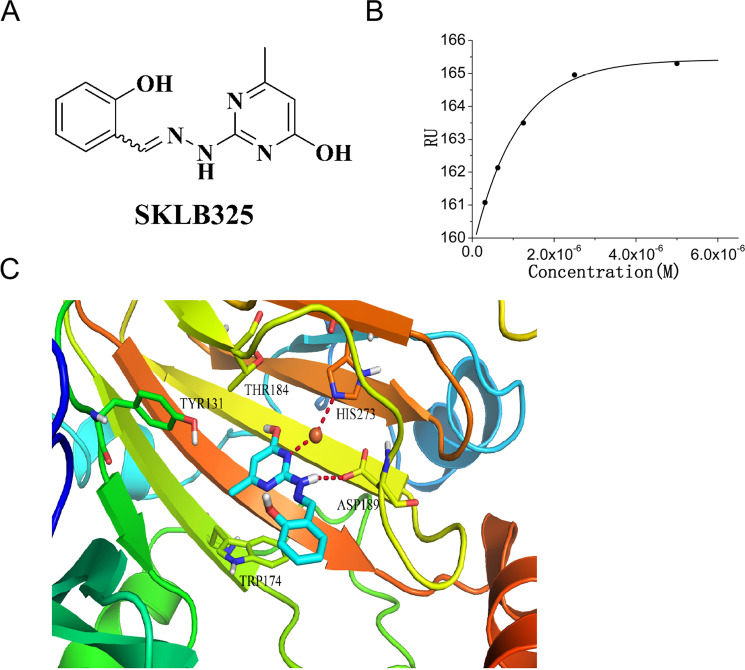
Identification and characterization of the inhibitor SKLB325. **a** Chemical structure of the inhibitor SKLB325. **b** Binding affinity of SKLB325 with JMJD6 by SPR assays. The concentrations of SKLB325 injected over the biosensor chip surface immobilized with JMJD6 protein are indicated. The measurement yielded a KD of 0.755 μM. **c** Predicted binding mode of SKLB325 in the active pocket of JMJD6. SKLB325 is color coded with a carbon atom in cyan, a nitrogen atom in blue, and an oxygen atom in red. Fe(II) is in orange. Coordination and hydrogen bonds are shown in the red dashed line

As JMJD6 was highly expressed in vitro and in vivo, as well as in HUVECs, which was examined by western blot and immunohistochemistry (Fig. 2a, b), we designed inhibitors of this molecule. To identify new inhibitors of JMJD6, we first screened compounds from several libraries according to a previous approach.^46^ The crystal structure of the jmjC domain of JMJD6 (PDB entry: 3LDB) was used to prepare the receptor structure for molecular docking. The original ligand was large enough to cover all the catalytic site, and the sphere containing the residues that stayed within 8 Å represented the active site; here, the active site is actually the binding site of α-ketoglutaric acid (α-OG). The Charmm force field was assigned. The GOLD program was used for molecular docking, and Goldscore was chosen to rank the compounds. We finally selected 20 agents from the top ranked compounds to carry out further experimental validation. One compound, namely, 2-(2-(2-hydroxybenzylidene) hydrazinyl)-6-methylpyrimidin-4-ol (termed SKLB325, Fig. 3a), was the most active one and showed a binding affinity (*K*_D_) value of 0.755 μM (Fig. 3b), and the IC_50_ value of the biochemical potency of the inhibitor SKLB325 was 0.7797 μM (Supplementary Fig. 1).

### Predicted binding mode of SKLB325 with JMJD6

The binding mode of SKLB325 in the active pocket of JMJD6 was predicted by molecular docking. Figure 3c shows a possible binding mode of SKLB325 with JMJD6. SKLB325 is suitably located in the binding site of α-OG. A strong coordination interaction is formed between the pyrimidine nitrogen (3-N) and Fe(II). In addition, the 2-position amino group forms a hydrogen bond with Asp189.

### JMJD6 inhibition suppresses proliferation, induces apoptosis in ovarian cancer cells, inhibits angiogenesis in *HUVECs*, and inhibits migration in ovarian cancer cells and HUVECs in vitro

**Fig. 4:**
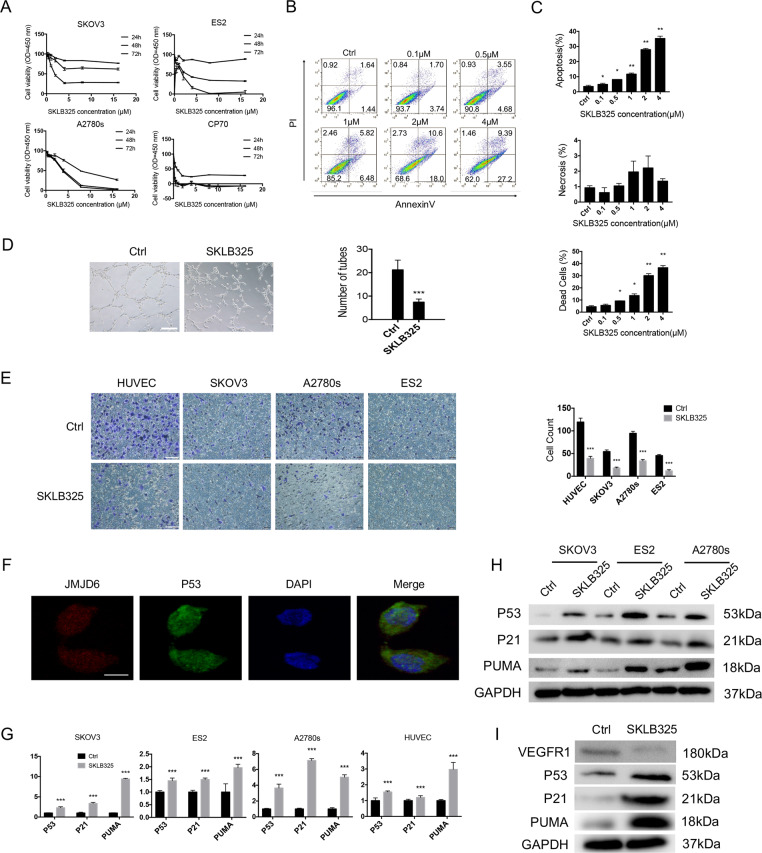
Antitumor effects of SKLB325 in vitro. **a** The effect of SKLB325 on the proliferation of SKOV3, ES2, A2780s and CP70 cells was determined by CCK-8 assays. Absorbances (450 nm) of cells cultured for 24 h, 48 h, and 72 h in the presence of various concentrations of SKLB325 (0, 0.25, 0.5, 1, 2, 4, 8, and 16 μM) were determined. Each drug concentration was tested in quadruplicate. The cell viability data (mean ± SD) are presented as percentages calculated by the following formula: Viability = 1- (SKLB325-vehicle)/(control-blank). **b**, **c** Representative analyses of apoptotic, necrotic and dead cells induced by various concentrations (0.1, 0.5, 1, 2, 4 μM) of SKLB325 in vitro were performed with flow cytometry with Annexin-V and PI staining. SKOV3 cells were treated with SKLB325 for 72 h. Percentages of apoptotic, necrotic and dead cells are shown. **d** HUVECs were seeded in 24-well culture plates precoated with Matrigel. Magnification: ×100, scale bar, 100 μm. **e** Migration of HUVECs, SKOV3, A2780s and ES2 cells was determined in chambers. Magnification: ×100, scale bar, 100 μm. **f** JMJD6 is physically associated with p53 in vitro. Immunofluorescence-stained endogenous JMJD6 (red) and p53 (green) were visualized by confocal microscopy. DAPI staining was included to visualize the cell nucleus (blue). Scale bar, 50 μm. **g** Measurement of mRNA levels of p53, p21 and PUMA by real-time RT-PCR. RT-PCR of cDNA obtained from SKOV3, ES2, A2780s, and HUVECs treated with or without SKLB325 for 72 h. **h** Measurement of p53, p21 and PUMA protein levels by western blotting. Western blot of protein abstracts obtained from SKOV3, ES2 and A2780s cells treated with or without SKLB325 for 72 h. **i** VEGFR1 protein levels were significantly downregulated after treatment with SKLB325 in HUVECs. p53, p21 and PUMA protein levels were significantly upregulated in HUVECs. The SKLB325 concentration was 4 μM. Data are presented as the mean values and standard deviations (SD). Each bar represents the mean ± SD for quadruple or triplicate experiments. For statistical comparisons, each treated group was compared with the control groups using Student’s *t* test. *p < 0.05, **p < 0.01

To explore the inhibitory effects on in vitro proliferation of ovarian cancer cells, CCK-8 was used to investigate the proliferation of ovarian cancer cells. As shown in Fig. 4a, with increasing SKLB325 concentration, the inhibitory effect also increased, exhibiting a significant dose-response relationship. There was a significant difference between the drug group across different doses and the control group (*p* < 0.05). These results indicated that SKLB325 had significant inhibitory effects on the in vitro proliferation of ovarian cancer cells. Furthermore, the most effective concentration at which JMJD6 inhibited SKOV3 cell growth was 4 μM, and the optimal duration of action was 72 h.

We next detected whether SKLB325 affected cell apoptosis. SKOV3, ES2 and A2780s cells treated with various concentrations were analyzed by PI and annexin V double staining. The same dose of DMSO according to the concentration of 4 μM was used in the control group. The results indicated that apoptotic cells observably increased (35.27 ± 1.56%, 28.39 ± 1.19%) in response to 4 μM treatment compared with those in the control group (3.57 ± 0.74%, 4.32 ± 1.09%, Fig. 4b and Supplementary Fig. 2A). Additionally, with increasing SKLB325 concentration, the percentage of apoptotic cells increased, suggesting that JMJD6 inhibition significantly promoted apoptosis in a dose-dependent manner. There were no apparent differences between various concentrations of SKLB325 in the levels of necrotic cells. However, the percentage of dead cells (sum of apoptotic cells and necrotic cells) increased (36.63 ± 1.22% versus 4.52 ± 0.60%, 32.17 ± 1.33% versus 3.78 ± 0.48%, 21.00 ± 0.01% versus 7.79 ± 1.08%, *p < 0.05, **p < 0.01, ***p < 0.001, Fig. 4c and Supplementary Fig. 2A and 2B). The results indicated that SKLB325 significantly induced the death of ovarian cancer cells. We also detected whether SKLB325 affected apoptosis or death in HUVECs. As Supplementary Fig. 2C shows, there were no differences between various concentrations of SKLB325 in the levels of apoptotic and dead cells in HUVECs.

To investigate the effect of SKLB325 on in vitro angiogenesis, we focused on the functions of SKLB325 in endothelial cells (ECs). As shown in Fig. 4d, the quantification of capillary tubes showed a 65.09% (21.2 ± 1.86% versus 7.4 ± 0.6%, ***p < 0.001) decrease at 4 h when HUVECs were seeded with 4 µM of the inhibitor simultaneously. Furthermore, transwell migration assays were used to assess the migration capacity of HUVECs treated with SKLB325. As shown in Fig. 4e, the number of HUVECs was 67.00% (119.4 ± 8.68% versus 39.4 ± 4.39%, ***p < 0.001) reduced following 4 µM inhibitor treatment for 12 h in the transwell migration assay. Similarly, the numbers of SKOV3, A2780s and ES2 cells showed 66.91% (54.4 ± 3.58% versus 18.0 ± 2.00%, ***p < 0.001), 64.27% (94.6 ± 4.51% versus 33.8 ± 3.03%, ***p < 0.001) and 74.12% (45.6 ± 2.30% versus 11.8 ± 2.59%, ***p < 0.001) reductions following 4 µM inhibitor treatment for 12 h in the transwell migration assay. These observations suggested that SKLB325 inhibited capillary tube organization and disrupted preformed capillary tubes by HUVECs on Matrigel, as well as inhibited the migration of ECs and ovarian cancer cells.

### SKLB325 upregulates the expression of p53 and its downstream effectors at both the mRNA and protein levels in vitro

To explore the cellular functions of JMJD6, confocal microscopy was used to determine the subcellular localization of endogenous JMJD6 and p53 in SKOV3 cells. The results demonstrated that p53 protein colocalized with JMJD6 in the nucleus (Fig. 4f).

To evaluate the expression of p53 as well as its downstream effectors in response to treatment with SKLB325 in vitro, SKOV3 cells were collected 72 h after exposure to SKLB325 or the same dose of DMSO and analyzed by qRT-PCR and western blots. The results showed that compared with the control group, the expression of p53, p21, and PUMA in SKOV3 cells was increased by 130.24%, 237.15%, and 841.36% in the SKLB325 group, respectively (***p < 0.001). Similarly, the expression of p53, p21, and PUMA in ES2 cells was increased by 45.08%, 49.92%, and 96.48% in the SKLB325 group (***p < 0.001), respectively. The expression of p53, p21, and PUMA in A2780s cells was increased by 265.10%, 613.73%, and 401.70% in the SKLB325 group, respectively (***p < 0.001). Furthermore, the expression of p53, p21, and PUMA in HUVECs was increased by 54.96%, 20.04%, 197.33% in the SKLB325 group (***p < 0.001) (Fig. 4g), respectively. As shown in Fig. 4h, p53, p21, and PUMA protein levels were significantly upregulated after treatment with SKLB325 in SKOV3, ES2 and A2780s cells. As shown in Fig. 4i, VEGFR1 protein levels were significantly downregulated after treatment with SKLB325 in HUVECs. However, p53, p21, and PUMA protein levels were significantly upregulated in HUVECs. The results of the western blot analysis were consistent with the results observed by RT-PCR. In general, these results indicated that p53 and its downstream effectors were effectively upregulated at both the mRNA and protein levels after treatment with SKLB325 in vitro.

### SKLB325 had antitumor activities in an intraperitoneal xenograft model

**Fig. 5:**
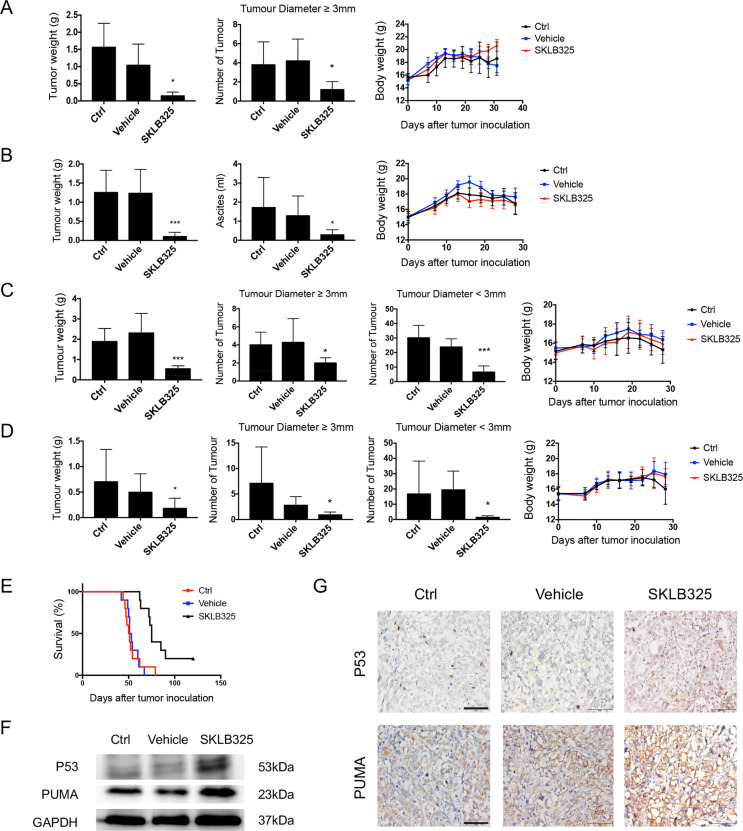
SKLB325 has strong therapeutic effects on intraperitoneal dissemination xenograft mouse models. **a** SKLB325 has strong therapeutic effects on a SKOV3 intraperitoneal dissemination xenograft mouse model: Intraperitoneal tumor weight of each group; number of tumors≥3 mm of each group; mice treated with SKLB325 showed no toxicity-dependent weight loss. **b** SKLB325 has strong therapeutic effects on an ES2 intraperitoneal dissemination xenograft mouse model: Ascites volume of each group; intraperitoneal tumor weight of each group; mice treated with SKLB325 showed no toxicity-dependent weight loss. **c** SKLB325 has strong therapeutic effects on a CP70 intraperitoneal dissemination xenograft mouse model: Number of tumours that were ≥3 mm in each group; Number of tumours that were ≤3 mm in each group; Intraperitoneal tumor weight of each group; Mice treated with SKLB325 showed no toxicity-dependent weight loss. **d** SKLB325 has strong therapeutic effects on an A2780s intraperitoneal dissemination xenograft mouse model: Number of tumors that were ≥3 mm in each group; Number of tumors that were ≤3 mm in each group; Intraperitoneal tumor weight of each group; Mice treated with SKLB325 showed no toxicity-dependent weight loss. Values are the mean ± SD; *n* = 5–7 mice/group. *p < 0.05, **p < 0.01, ***p < 0.001, SKLB325 group versus control and vehicle groups, respectively. Control: without any treatment; Vehicle: the same dose of DMSO accompanied with NS. **e** Kaplan–Meier survival curve for tumor-bearing mice treated with control, vehicle, or SKLB325 using the SKOV3 intraperitoneal dissemination xenograft mouse model. Two mice in the SKLB325-treated group survived the entire 120 days after i.p. inoculation, when the planned experimental period ended. Survival was significantly longer in the mice treated with SKLB325 compared with the control and vehicle groups (log-rank test, *p* = 0.000), and there was no significant difference between the control and vehicle groups (*p* > 0.05). The median survival of the SKLB325 group was 75 days versus 51 days and 52 days in the control and vehicle groups, respectively; *n* = 10 for each group. **f** Measurement of protein levels of P53 and PUMA by western blotting in vivo on the SKOV3 intraperitoneal dissemination xenograft mouse model. **g** Immunohistochemistry of p53 and PUMA in tumor tissue in the SKOV3 intraperitoneal dissemination xenograft mouse model. Magnification: ×200; scale bar, 50 μm

Then, we performed comparative analyses of JMJD6 and catalytic domain (JMJC) proteins of human and mouse; as shown in Supplementary Fig. 3A and B, mouse JMJD6 displays 98% (403 aa) identity with human JMJD6 and mouse JMJC displays 99% (165 aa) identity with human JMJC. The results demonstrated that it was reasonable to use the mouse model to explore the effects and side effects of SKLB325. As SKLB325 could induce apoptosis and inhibit cell proliferation, as shown above, this inhibitor had antitumor potential in vivo. Therefore, we established an intraperitoneal xenograft model to further evaluate the effect of SKLB325 in vivo. Tumor nodules in the two control groups were scattered on the kidney, pancreas, or parenchyma of the liver. In the SKLB325 group, the growth of intraperitoneal tumor nodules was limited to the pelvis, and organ and tissue invasion was absent. Tumor nodules varied in size, but they were more numerous and larger in the two control groups than in the SKLB325 group. The average weight of intraperitoneal tumor nodules was 1.56 ± 0.70, 1.04 ± 0.62, and 0.14 ± 0.11 g in the control, vehicle and SKLB325 groups, respectively. Tumor weight was significantly reduced by 91 and 86% in the SKLB325 groups compared to the control and vehicle groups, respectively (*p* < 0.05, Fig. 5a). Compared to treatment with control or vehicle, treatment with SKLB325 significantly suppressed tumor growth, while no significant difference was observed in tumor weight between the control and vehicle groups (*p* = 0.108). The number of tumors whose diameter was ≥3 mm in the SKLB325 group was reduced compared to the two control groups (Fig. 5a). Similar results are shown in Fig. 5b–d. In the face of limitations by institutional ethics, the results of an animal study still indicated that the SKLB325 treatment protocols were effective in suppressing SKOV3, ES2, CP70, and A2780s tumor growth in nude mice.

There were no abnormalities visible to the naked eye observed in mice treated with SKLB325. No significant differences in body weight were found among the three groups (Fig. 5a–d). In addition to reduced cell proliferation and increased apoptosis, survival time also reflected the beneficial effects of SKLB325 on this human ovarian cancer model. As shown in the Kaplan–Meier survival curve in Fig. 5e, mice in the SKLB325 group lived longer than those in the two control groups, with a median survival of 75 days compared to 51 days and 52 days. No statistically significant difference in survival was observed between the control and vehicle groups (*p* > 0.05). At 120 days after i.p. inoculation, two mice in the SKLB325-treated group still survived when the experiment ended. These results demonstrated significant advantages in the SKLB325-treated group (*p* < 0.001).

### SKLB325 upregulates the expression of p53 as well as its downstream effectors at the protein level in vivo

To detect the expression of p53 and its downstream effectors, western blotting was performed. As shown in Fig. 5f, the protein expression of p53 and PUMA was increased. Immunohistochemical staining in tumor tissues further demonstrated the promotion of p53 and PUMA in the inhibitor-treated tumours, as shown in Fig. 5g, Supplementary Figs. 4 and 5. Together, these results indicated that p53 and PUMA were induced after treatment with SKLB325 in vivo.

### SKLB325 inhibits cell proliferation and induces cell apoptosis in vivo

**Fig. 6:**
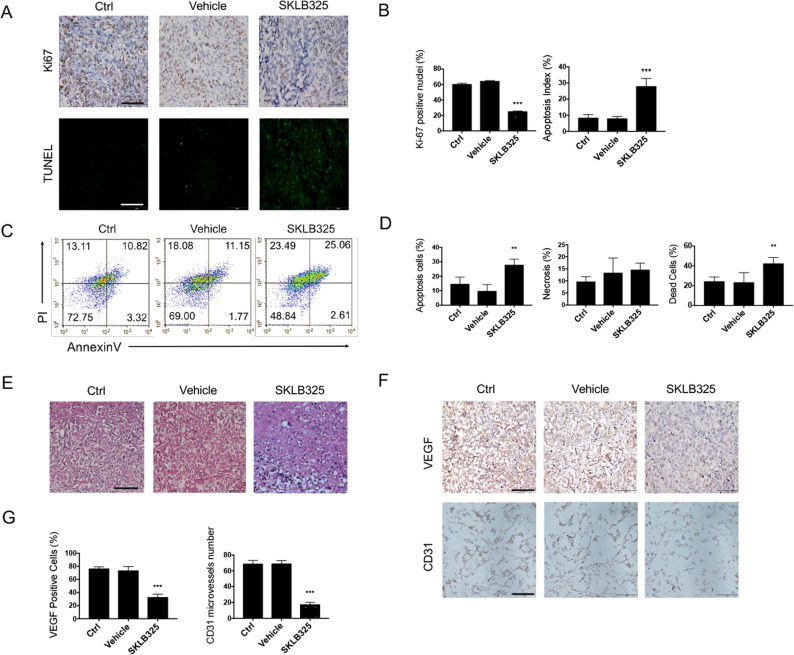
SKLB325 induces apoptosis and suppresses angiogenesis in vivo. **a** Representative images for immunohistochemical staining of Ki-67 and TUNEL on SKOV3 tumors isolated from mice treated with control, vehicle and SKLB325. The data showed that SKLB325 can lead to suppression of tumor cell proliferative activity (scale bar, 50 μm) and promotion of tumor apoptotic activity (scale bar, 100 μm). **b** Percentages of Ki-67-positive nuclei apparently decreased, and percentages of TUNEL-positive nuclei apparently increased. The number of Ki-67-positive or TUNEL-positive cells was counted in five different fields under a light microscope at ×200 magnification, ***p < 0.001. The results are presented as the mean ± SD (*n* = 5) versus control and vehicle groups. **c** Representative images for flow cytometry with Annexin-V and PI staining of peritoneal lavage fluid showed intraperitoneal apoptotic cells increased in the mice that were treated with SKLB325. **d** Percentages of total apoptotic cells, necrotic and dead cells are shown. Each bar represents the mean ± SD (*n* = 5); **p < 0.01. **e** Representative images of hematoxylin and eosin (H & E) staining of intraperitoneal tumours isolated from mice treated with control, vehicle or SKLB325. No obvious necrotic cells were found in the tumors isolated from mice with control or vehicle treatment, but tumours isolated from mice with SKLB325 treatment exhibited large areas of necrotic or apoptotic cells. Magnification: ×100; scale bar, 100 μm. **f** Representative images of immunohistochemical VEGF and CD31 staining. Magnification: ×100; scale bar, 100 μm. The quantification of MVD was estimated by counting the number of microvessels in five random fields at ×200 magnification. **g** Immunostaining of VEGF on tumor tissue. A single positively stained cell or single microvessel was defined as a discrete cluster for CD31. The SKLB325-treated group showed reduced stained cells and microvessel density. Data are presented as the mean ± SD (*n* = 5). ***p < 0.001 versus the other two groups; control group: without any treatment; vehicle group: the same dose of DMSO accompanied with NS

To further confirm cell proliferation and apoptosis in vivo, we measured their effects by Ki-67 and TUNEL staining, as well as flow cytometry with Annexin-V and PI staining. The percentage of Ki-67-positive cells was significantly reduced in the SKLB325 group compared to the control and vehicle groups (25.11 ± 1.27 versus 60.20 ± 1.66 and 64.19 ± 1.09, respectively, ***p < 0.001, Fig. 6a, b). Many strongly positive nuclei were found in SKLB325-treated tumor tissues, as determined by immunofluorescence microscopy of TUNEL staining; however, such nuclei were rare in tumor tissues of the control groups (28.23 ± 5.07 versus 8.90 ± 2.19 and 7.28 ± 1.52, respectively, ***p < 0.001, Fig. 6a, b). Furthermore, flow cytometry with Annexin-V and PI staining was used to investigate apoptosis of intraperitoneal cells in peritoneal lavage fluid. The results showed that intraperitoneal apoptotic cells (27.62 ± 1.90% versus 14.45 ± 2.21% and 9.59 ± 2.12%, respectively, **p < 0.01, Fig. 6c) and dead cells (42.08 ± 2.82% versus 24.00 ± 2.15% and 22.97 ± 4.60%, respectively, **p < 0.01, Fig. 6d) were remarkably increased in mice treated with SKLB325 compared to the control and vehicle groups. As shown in Fig. 6e, in histopathological examination of tumor tissues stained with H&E, we detected no obvious necrotic or apoptotic cells in the tumours isolated from mice with control or vehicle treatment, but tumours isolated from mice in the SKLB325 group exhibited large areas of necrotic or apoptotic cells.

### Inhibition of angiogenesis by SKLB325 in vivo

As the antiangiogenic effect of SKLB325 was found in vitro, we used immunohistochemical staining of VEGF and CD31, which had highly specific affinity for vascular endothelial cells, for further study. We measured microvessel numbers in endothelial cells that were stained by VEGF and CD31 and evaluated tumor sections for VEGF and CD31 staining. The number of cells stained by VEGF was significantly reduced in tumors treated with SKLB325 (17.10 ± 2.21) compared with the control and vehicle groups (40.97 ± 1.13 and 49.49 ± 1.56, respectively, ***p < 0.001, Fig. 6f, g). Similarly, the number of microvessels marked by CD31 was also significantly reduced in tumours treated with SKLB325 (17.00 ± 1.41) compared with the two control groups (68.40 ± 2.14 and 68.60 ± 1.97, ***p < 0.001, Fig. 6f, g). These results demonstrated that angiogenesis within this intraperitoneal carcinomatosis model was inhibited in these mice of the SKLB325 group, suggesting that the antitumor activity of the inhibitor was due, at least in part, to inhibition of angiogenesis in our tumor model.

### No noticeable adverse effects were found in the intraperitoneal dissemination xenograft mouse mode

**Fig. 7:**
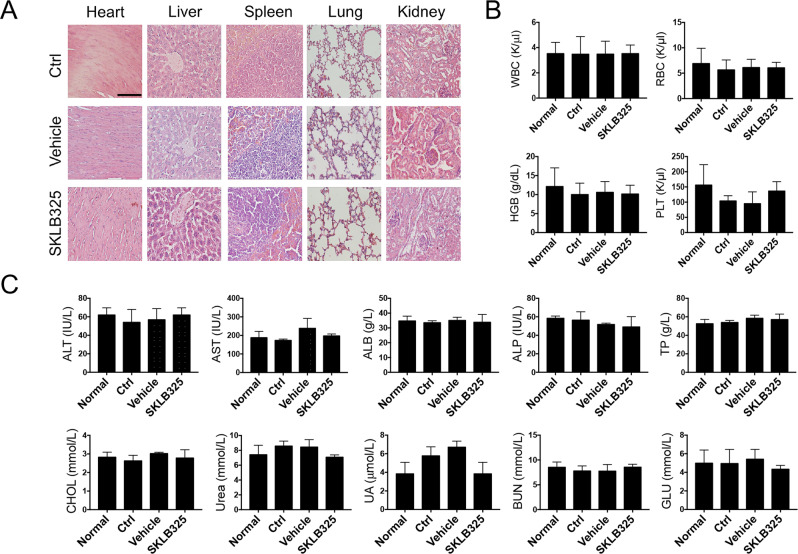
No noticeable adverse effects were found in the intraperitoneal dissemination xenograft mouse model. **a** Histological staining of heart, liver, spleen, lung, and kidney from mice treated with SKLB325 revealed no significant pathologic differences (*n* = 5). Magnification: ×200; scale bar, 50 μm. **b** The results of WBC, RBC, HGB and PLT in the normal group, control group, vehicle group and SKLB325 group exhibited no significant differences among these four groups. **c** Results of ALT, AST, ALB, ALP, TP, CHOL, urea, UA, BUN, and GLU among the normal, control, vehicle and SKLB325 groups exhibited no significant differences in these four groups

Histological examination of the heart, spleen, kidney, lung, and liver revealed no significant differences among all groups (Fig. 7a). These results further indicated that SKLB325 treatment did not cause obvious adverse effects at the indicated doses and times in vivo, and the mice showed good tolerance of the current treatment procedures. Routine analysis and biochemical analysis of blood from all experimental groups and a normal group with no treatment were collected. White blood cells (WBC), red blood cells (RBC), hemoglobin (HGB), and platelets (PLT) exhibited no significant differences among these four groups (Fig. 7b). Furthermore, there were no significant differences in alanine transaminase (ALT), aspartate transaminase (AST), albumin (ALB), alkaline phosphatase (ALP), total protein (TP), cholesterol (CHOL), urea, uric acid (UA), blood urea nitrogen (BUN), or glucose (GLU) among the normal, control, vehicle, and SKLB325 groups (Fig. 7c).

## Discussion

Mortality of ovarian cancer remains extraordinarily high, despite many improvements in treatments for patients with ovarian cancer, including surgery, radiotherapy, chemotherapy, and new biological therapies.^5–7^ Furthermore, 60–70% of patients with ovarian cancer who respond to standard primary treatment relapse or die within 5 years after initial diagnosis.^8,47^ This terrible situation has stimulated the development of new strategies to improve therapeutic efficacy.

Recent studies indicate that ablation of the JMJD6 gene in *C. elegans* delays the engulfment of apoptotic cells,^15,37^ and depletion of JMJD6 promoted apoptosis, induced cell death by sensitizing cells to DNA damaging reagent, and repressed cell tumorigenesis and proliferation, which is mediated by p53.^9^ The expression of JMJD6 in some human cancers is evidently upregulated, such as lung adenocarcinoma,^39^ breast ductal carcinomas,^40^ and colon adenocarcinomas.^9^ Furthermore, high expression of JMJD6 is related to tumor growth, tumor metastasis, and high tumor pathological classification,^9,38–41^ indicating that JMJD6 may act as a novel therapeutic target for cancer intervention. However, there is still limited information on the expression of JMJD6 in ovarian cancer. Furthermore, no JMJD6 inhibitors have been developed and applied to the research of ovarian cancer.

In our research, JMJD6 protein expression was detected by immunohistochemical staining in a cohort of 146 ovarian cancer specimens, and we analyzed the relationship between JMJD6 protein expression and clinicopathological variables. The results indicated that high expression of JMJD6 in ovarian cancer was significantly associated with age, clinical stage, pT status, pN status, and relapse. Patients with high JMJD6 expression had significantly worse OS and DFS. These results clearly demonstrated that high expression of JMJD6 was associated with adverse clinical outcomes of ovarian cancer. In view of the above results, we demonstrated that JMJD6 is important in ovarian cancer and may provide a novel therapeutic target.

Our study investigated whether SKLB325 inhibition could be a novel therapeutic approach to combat ovarian cancer by repressing cell proliferation and tumorigenesis. Several observations have been made in this study concerning the antitumor effects of SKLB325 on ovarian cancer. The inhibitor SKLB325 exerted a cytostatic effect, the results of the CCK-8 cell proliferation assay indicated that SKLB325 could suppress cell proliferation in vitro, and this treatment led to inhibition of cell proliferation in a significant dose-dependent manner. Furthermore, establishment of a peritoneal carcinomatosis model in BALB/c nude mice provided evidence that JMJD6 inhibition had strong therapeutic effects on an intraperitoneal dissemination xenograft mouse model. Tumor volumes in the SKLB325 group were notably reduced compared with the two control groups, which was in agreement with extensive reports that have supported the role of JMJD6 in carcinogenesis.^9,39^ SKLB325 prolonged the survival of tumor-bearing mice. The results showed no noticeable adverse effects in the intraperitoneal dissemination xenograft mouse mode and provided evidence for the safety of the inhibitor SKLB325.

Many studies have shown that inducing apoptosis and inhibiting angiogenesis are possible mechanisms of the inhibitor SKLB325.^9^ Our flow cytometry and PI/Annexin V results indicated that the inhibitor SKLB325 induced apoptosis and cell death. Recent studies showing that JMJD6 could increase proliferation in MCF-7 cells (breast adenocarcinoma cells) also supported our results.^40^ Angiogenesis is a key factor in the development of metastatic carcinoma,^48^ and many previous studies have proven that angiogenesis is necessary for tumorigenesis and progression,^49–51^ which is required for tumor formation, as well as for delivering oxygen and nutrients.^52^ CD31 and VEGF, markers of angiogenesis, have been identified as prognostic indicators in ovarian cancer.^53–57^ The Matrigel endothelial tube formation assay suggested that SKLB325 could contribute to inhibiting angiogenesis in HUVECs in vitro, which was consistent with a previous study showing that JMJD6 silencing could affect the splicing of angiogenesis-related genes (VEGF receptor 1) in ECs and that it was necessary for angiogenic sprouting.^58^ Furthermore, we found that p53 protein colocalized with JMJD6 in the nucleus and that SKLB325 could upregulate p53 and the expression of downstream effectors at both the mRNA and protein levels in vitro. Similar to previous research, JMJD6 had a negative effect on the p53 pathway, and depletion of JMJD6 promoted apoptosis, induced cell death, and repressed cell tumorigenesis and proliferation, which is mediated by p53.^9^ Consistent with the results in vitro, staining of Ki-67, TUNEL analysis and flow cytometry with Annexin-V and PI indicated that SKLB325 could inhibit cell proliferation and induce cell apoptosis in vivo. Immunohistochemical staining also suggested that SKLB325 could upregulate p53 and its downstream effectors. We found inhibition of angiogenesis by SKLB325 in vivo as detected by immunohistochemical staining of VEGF and CD31, which coincided with previous studies.^58^ Although no studies on the antitumor effects of JMJD6 inhibitors have been published, previous studies have reported that tumor growth was obviously impaired in BALB/c nude mice that received JMJD6-depleted HCT116 p53+/+tumor transplants, and the literature supports the probability that inhibition of JMJD6 may have antitumor effects on human cancers.^9^ However, there are still some shortcomings in our experiment; for example, JMJD6 depletion or p53 depletion experiments may confirm that SKLB325 suppresses cancer progression through inhibition of JMJD6 and upregulation of p53 expression. In addition, the inhibition of the enzymatic activity of JMJD6 and other family members of JmjC domain-containing proteins by the inhibitor SKLB325 should be performed in future studies.

## Conclusions

In the present study, we demonstrated that JMJD6 may be a marker of poor prognosis in ovarian cancer. Furthermore, we found that the inhibitor SKLB325 suppresses ovarian cancer growth through inhibition of proliferation and induction of apoptosis and cell death, and inhibiting angiogenesis may play a significant role in inhibiting tumor growth. SKLB325 significantly prolongs the survival of tumor-bearing mice without obvious side effects. These results suggest that SKLB325 may be a potential candidate drug for treatment of ovarian cancer.

## Supplementary information

Supplementary Figures.

## Data Availability

All data generated or analyzed during this study are included in this published article.
