# Supplementary material for: Jumonji domain-containing 6 (JMJD6) identified as a potential therapeutic target in ovarian cancer
Source: Signal Transduct Target Ther. 2019 Jul 26;4:24. doi: 10.1038/s41392-019-0055-8 (PMC6799828; doi:10.1038/s41392-019-0055-8)
Supplement: Supplementary file 1 — Supplementary Figures. [file 41392_2019_55_MOESM1_ESM.pdf]

Supplementary Information

Role of JMJD6 in Ovarian Cancer and Development of a Novel JMJD6 Inhibitor for the Potential Targeted Therapy

Heng Zheng[a,1], Yan Tie[b,1,], Zhen Fang[b], Xiaoai Wu[b], Tao Yi[a], Shuang Huang[a], Xiao Liang[a], Yanping Qian[a], Xi Wang[a], Ruyu Pi[a], Siyuan Chen[b], Yong Peng[b], Shengyong Yang[b], Yuquan Wei[b], Xia Zhao[a], Xiawei Wei[b, *]

[a] Department of Gynecology and Obstetrics, Key Laboratory of Obstetric and Gynecologic and Pediatric Diseases and Birth Defects of Ministry of Education, West China Second Hospital, Sichuan University, Chengdu,610041, P.R. China.

[b] Lab of Aging Research and Cancer Drug Targets, State Key Laboratory of Biotherapy, West China Hospital, Sichuan University and Collaborative Innovation Center, No. 17, Block 3, Southern Renmin Road, Chengdu, Sichuan 610041, P.R. China.

[1]These authors contributed equally to this work.

[*]Corresponding author: Xiawei Wei, xiaweiwei@scu.edu.cn

Lab of Aging Research and Cancer Drug Targets, State Key Laboratory of Biotherapy, West China Hospital, Sichuan University and Collaborative Innovation Center, No. 17, Block 3, Southern Renmin Road, Chengdu, Sichuan 610041, P.R. China.

E-mail addresses: xiaweiwei@scu.edu.cn

Tel.: +86 28 85502796; Fax: +86 28 85502796.

This file contains the Supplementary Figure1-5. Supplementary Fig. 1 showed SKLB325 inhibited enzymatic activity of JMJD6. And it showed the induction on cell apoptosis (Supplementary Fig. 2). As Supplementary Fig. 3 showed, the protein identity between human and mouse JMJD6 protein indicated that it was reasonable to use the mouse model to explore the effects and side effects of SKLB325. And Supplementary Fig. 4-5 showed the immunohistochemistry of p53 and PUMA in tumour tissue on ES2 and CP70 intraperitoneal dissemination xenograft mouse modes.

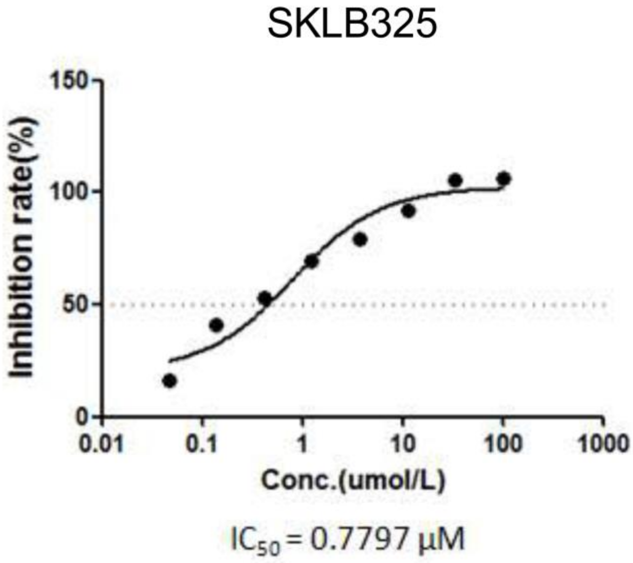

Supplementary Fig. 1 Biochemical potency of the inhibitor SKLB325, with an IC_{50} value of 0.7797μM.

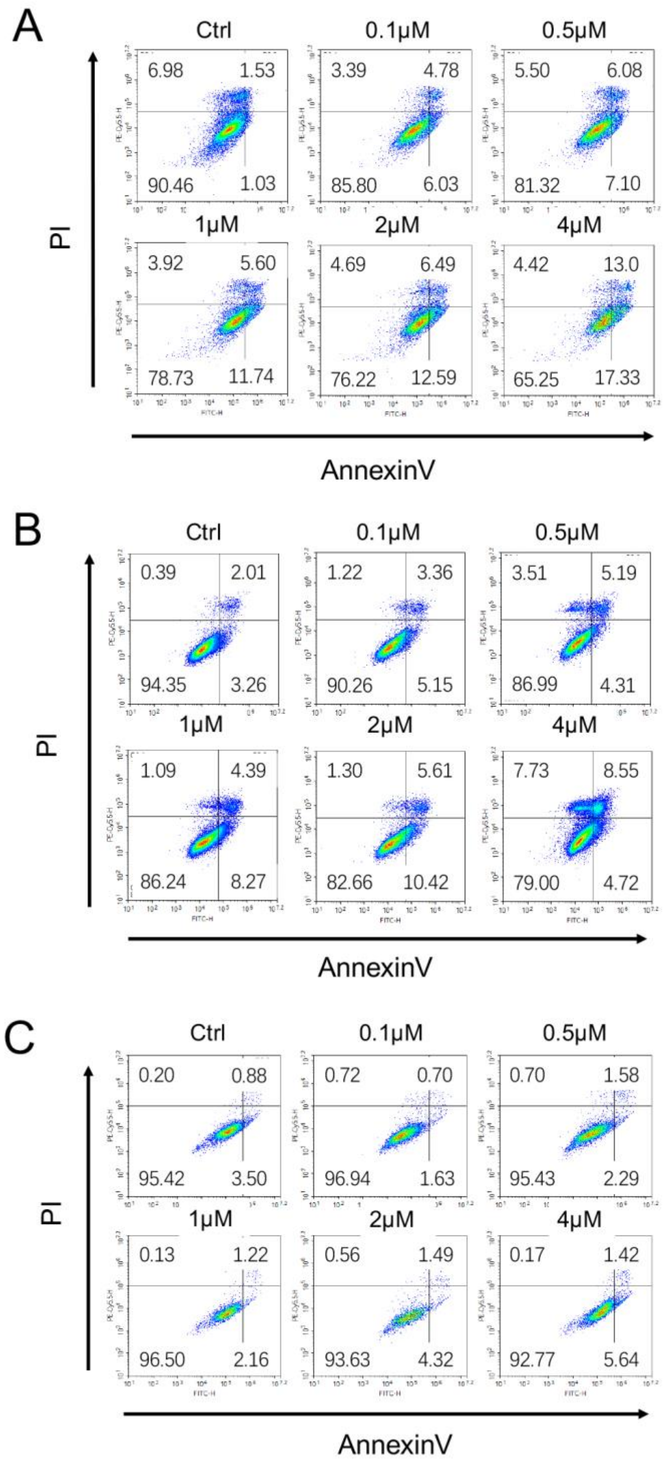

Supplementary Fig. 2 Representative experiment of apoptotic and dead cells induced by various concentrations (0.1, 0.5, 1, 2, 4 μM) of SKLB325 in vitro was detected by flow cytometry with Annexin-V and PI staining. **(A)** A2780s, **(B)** ES2 and **(C)** HUVECs were treated with SKLB325 for 72 h. Percentages of apoptotic and dead cells are shown.

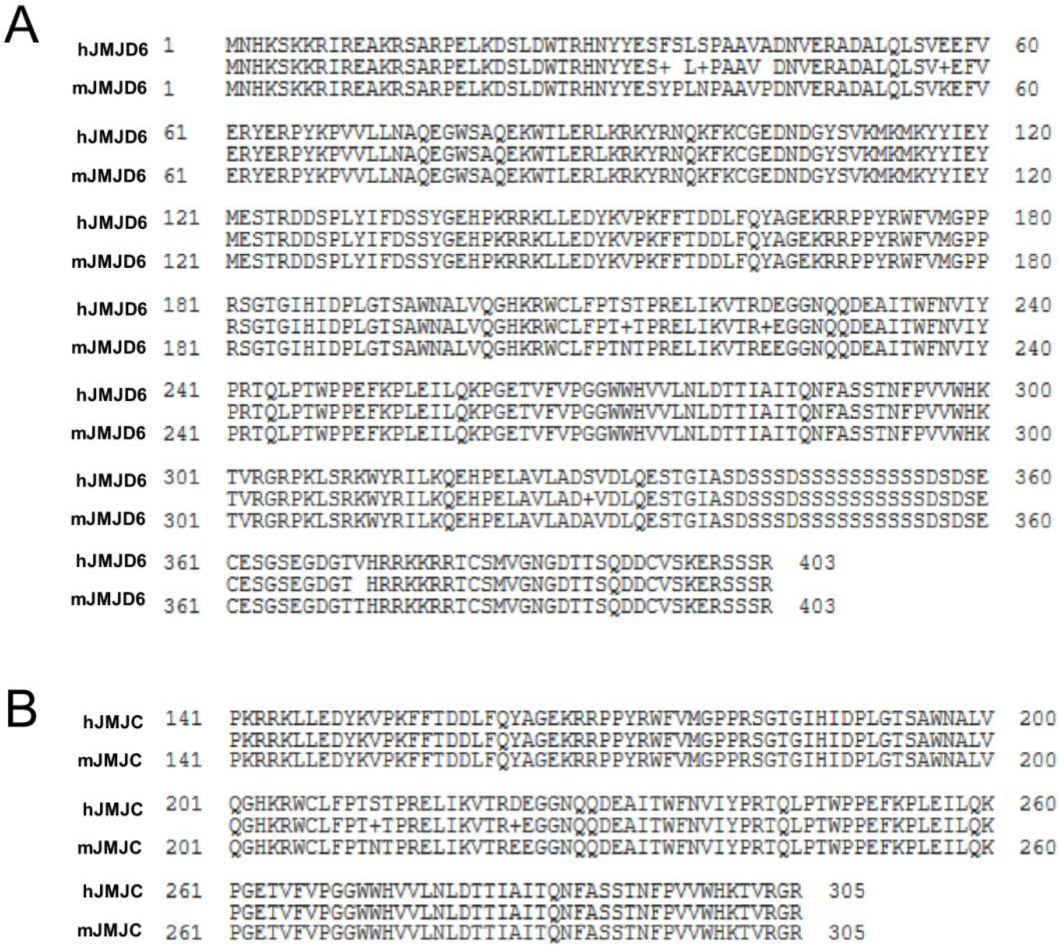

Supplementary Fig. 3 Protein alignments of human and mouse JMJD6 and catalytic domain (JMJC). (A) Mouse JMJD6 displays 98% (403aa) identity with human JMJD6. **(B)** Mouse JMJC displays 99% (165aa) identity with human JMJC.

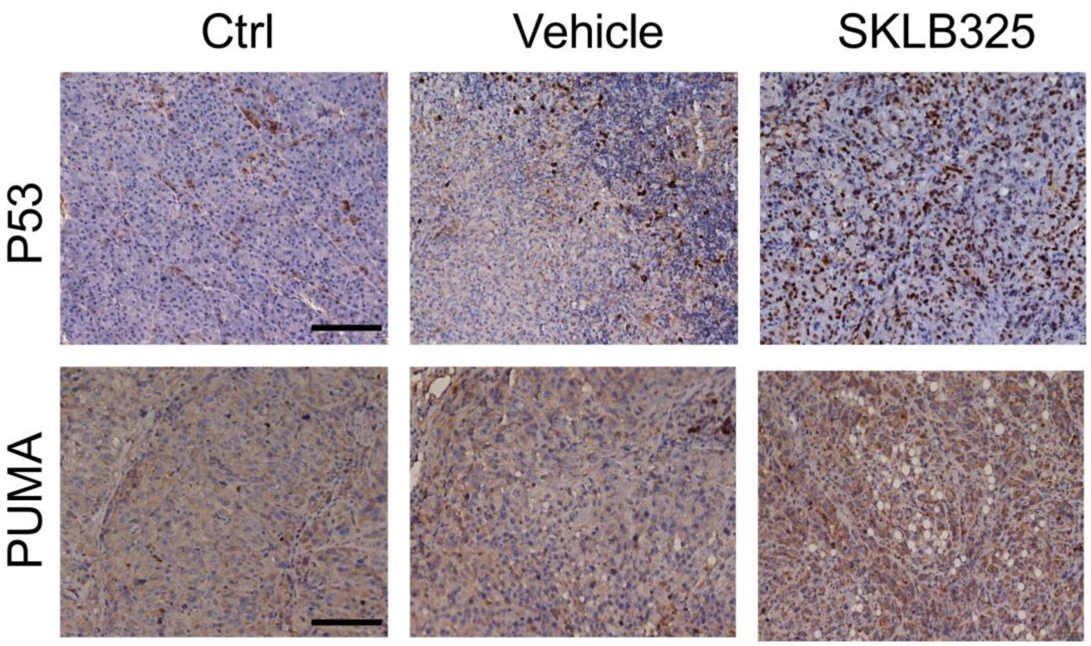

Supplementary Fig. 4 Immunohistochemistry of p53 and PUMA in tumour tissue on ES2 intraperitoneal dissemination xenograft mouse mode. Magnification: 200 ×; scale bar, 50 μm.

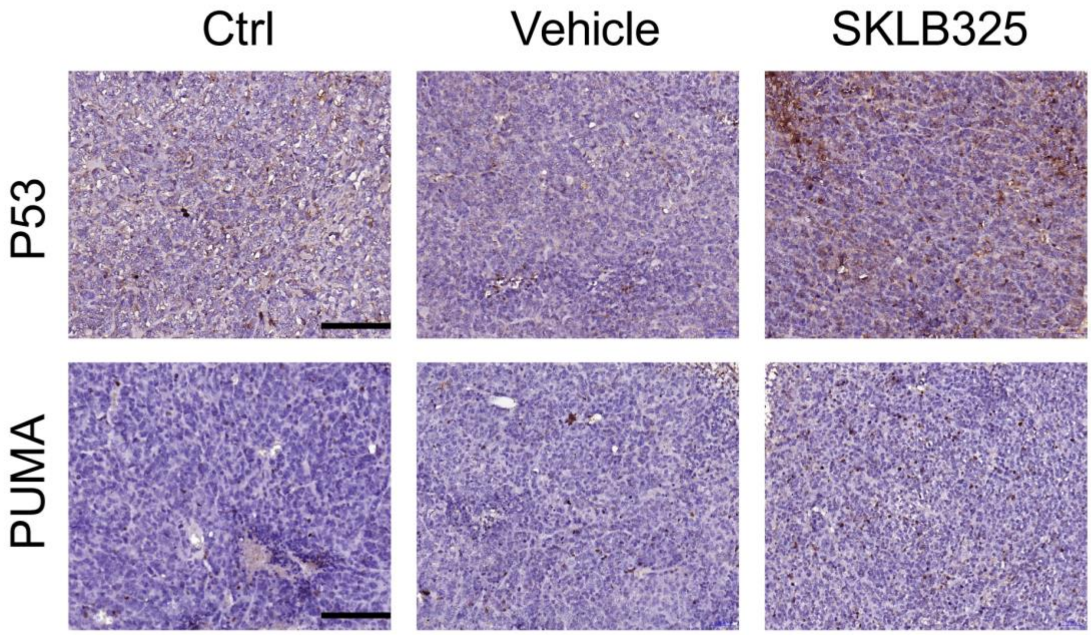

Supplementary Fig. 5 Immunohistochemistry of p53 and PUMA in tumour tissue on CP70 intraperitoneal dissemination xenograft mouse mode. Magnification: 200 ×; scale bar, 50 μm.
